# Total hip arthroplasty via the direct anterior approach with a dual mobility cup for displaced femoral neck fracture in patients with a high risk of dislocation

**DOI:** 10.1051/sicotj/2017048

**Published:** 2017-10-06

**Authors:** Hironori Ochi, Tomonori Baba, Yasuhiro Homma, Mikio Matsumoto, Taiji Watari, Yu Ozaki, Hideo Kobayashi, Kazuo Kaneko

**Affiliations:** 1 Department of Orthopedic Surgery, Juntendo University School of Medicine 2-1-1 Hongo Bunkyo-ku Tokyo 113-8421 Japan; 2 Department of Orthopedic Surgery, Sanikukai Hospital 3-20-2 Taihei Sumida-ku Tokyo 130-0012 Japan

**Keywords:** Displaced femoral neck fracture, Direct anterior approach, Total hip arthroplasty, Dual mobility cup, Single conventional cup

## Abstract

*Introduction*: Although total hip arthroplasty (THA) is superior to bipolar hemiarthroplasty (BHA) for displaced femoral neck fracture in terms of hip pain, function and reoperation rate, THA has a higher rate of dislocation. The direct anterior approach (DAA) and a dual mobility cup (DMC) are associated with lower rates of dislocation. The aim of this study was to investigate the outcomes of THA compared with BHA, and in those patients who had a THA we investigated those with a DMC (DMC-THA) and compared them with those had a single conventional cup (Single cup-THA).

*Materials*: A total of 89 patients living independently were included between 2009 and 2015. We assessed patient characteristics, peri- and post-operative outcomes, walking ability and one-year mortality. Adjusted odds ratios (Adjusted ORs) were estimated for decrease of walking ability and one-year mortality using a logistic regression model with adjustment for potential confounders such as age, neuromuscular diseases with weakness, duration of surgery, perioperative blood loss and preoperative walking ability.

*Results*: BHA (20 patients) versus THA (69 patients): There was no significant difference in the walking ability in either group. Multivariable logistic regression analysis demonstrated a significant association with one-year mortality in both groups [THA Adjusted ORs 0.088 (95% CI 0.0007–0.69); *p* = 0.020]. Single cup-THA (36 patients) versus DMC-THA (33 patients): The DMC-THA group had significantly greater age and more patients with neuromuscular diseases with weakness compared with the Single cup-THA group. Multivariable logistic regression analysis demonstrated no significant difference in the decrease of walking ability and in the one-year mortality between the groups. There were no post operative dislocations in any group.

*Discussion*: THA via the DAA is one of the best treatments for displaced femoral neck fracture with a low risk of dislocation. THA via the DAA with a DMC is a safe and effective treatment for the patients with a high risk of dislocation.

## Introduction

Displaced femoral neck fracture is generally treated via arthroplasty, as this treatment allows early mobilisation of the patient [1]. Bipolar hemiarthroplasty (BHA) and total hip arthroplasty (THA) are widely accepted methods of hip replacement after fracture. Although THA is reportedly superior to BHA in terms of hip pain, function and reoperation rate, THA has a higher rate of dislocation [2, 3]. The direct anterior approach (DAA) to the hip joint is an intermuscular and inter-neural approach without muscle transection and detachment [4, 5]. The DAA is gaining popularity because of its perceived earlier postoperative recovery and lower dislocation rate [4, 6, 7]. The DAA has proven beneficial in trauma patients, including those with femoral neck fracture [4, 8]. The dual mobility cup (DMC), developed by French surgeon Gilles Bousquet in the 1970s, is an implant with a mobile polyethylene liner and a greater range of motion compared with other implants [9–11]. THA with a DMC is associated with lower rates of dislocation in elective surgery and revision procedures compared to THA with a single conventional cup [1, 12, 13].

Several studies have compared BHA and THA for displaced femoral neck fracture [3, 14–16]. However, few previous reports have compared BHA via the DAA with THA via the DAA, or compared THA via the DAA with a single conventional cup with a DMC for displaced femoral neck fracture. We hypothesised that THA via the DAA with a DMC would be safe and effective treatments for displaced femoral neck fracture in patients with a high risk of dislocation. The aim of this study was to investigate the clinical outcomes of THA via the DAA compared with the outcomes of BHA via the DAA for displaced femoral neck fracture, and in those patients who had a THA to compare DMC (DMC-THA) with a single conventional cup (Single cup-THA). We wished to establish whether DMC-THA via the DAA is more effective for the patients with a high risk of dislocation.

## Materials and methods

### Subjects

Institutional Review Board approval was obtained before review of any medical records. The population consisted of three groups of patients who were all treated with primary arthroplasty for displaced femoral neck fracture in our two hospitals between November 2009 and June 2015. In the period between 2009 and 2013, the standard treatment for displaced femoral neck fracture in our hospital was cementless BHA via the DAA; all such surgeries in this period were performed by one senior surgeon (TB). During 2013–2015, the standard treatment for hip fracture was changed from BHA to THA via the DAA. During this period, patients underwent either Single cup-THA or DMC-THA in one of the two hospitals. For patients with displaced femoral neck fracture, the Single cup-THAs were performed in one hospital and the DMC-THAs were performed in the other hospital at random. From 2013, three adult reconstructive surgeons (MM, YH and HO) performed THA operations in conjunction with a DAA expert senior surgeon (TB) who participated in all surgeries in the two hospitals. Exclusion criteria were: (1) previous history of any osteotomy surgery on the ipsilateral side and (2) pathological fracture. However, there were no patients with such conditions in this study. A total of 89 patients living independently were included in this retrospective review.

### Evaluation

We retrospectively assessed patient characteristics including the presence of neuromuscular diseases with weakness such as cerebrovascular disorder and Parkinson’s disease [17], duration of surgery, intraoperative blood loss and intraoperative complications such as femur penetration of the implant and femoral shaft fracture. We also recorded postoperative complications including dislocation, deep infection, reoperation and one-year mortality. Walking ability was recorded preoperatively and one-year postoperatively. The walking ability was stratified into the following four categories: (1) unaided walking (this category included walking with a T-cane, as our hospital protocol is for patients to use a T-cane even when unaided walking is possible), (2) walking using two crutches (including walkers for the elderly), (3) walking alongside a support (assisted walking) and (4) use of a wheelchair [8]. A decrease of walking ability was defined as this category level decreasing by one point or more.

The first study compared BHA via the DAA (BHA group) with THA via the DAA (THA group), and the second study compared the Single cup-THA group with the DMC-THA group.

### Direct anterior approach technique with manual leg control and fluoroscopy

The DAA was performed as previously reported using the distal part of the Smith-Petersen approach with the patient in the supine position on a standard surgical table [4, 8, 18]. In THA cases, we adjusted the cup setup with a trial handle, aiming for an inclination angle of 40° and an anteversion angle of 25°. Intraoperative control of cup and shaft positioning was always performed by fluoroscopy and adjusted as needed to ensure optimal positioning. All patients were allowed to fully weight-bear immediately after their operation.

### Implant data

All patients received cementless tapered-wedge femoral components with circumferential plasma spray coating and hydroxyapatite. The stem used was the Profemur^®^ TL (Wright Medical Technology, Memphis, TN, USA) in 25 hips, the Accolade TMZF^®^ (Stryker Orthopaedics, Mahwah, NJ, USA) in 52 hips and the TriLock^®^ (DePuy Orthopaedics, Warsaw, IN) in 12 hips. In the BHA group, the prosthetic head size was available in 2-mm increments that allowed accurate reproduction of each patient’s femoral head, which was measured intraoperatively with a hemispherical template. All patients received cementless cups. In the Single-THA group, the diameter of the inner head was 32 mm in 33 hips and 36 mm in three hips; the acetabular cup used was the Trident^®^ acetabular shell (Stryker Orthopaedics, Mahwah, NJ, USA) in four hips, the Nakashima THA Cup (Nakashima Medical Co. Ltd, Okayama, Japan) in 13 hips, the Dynasty^®^ Biofoam^TM^ Acetabular Cup System (Wright Medical Technology, Memphis, TN, USA) in seven hips and the Pinnacle^®^ Acetabular Cup System (DePuy Orthopaedics, Warsaw, IN) in 12 hips. In the DMC-THA group, the Trident^®^ hemispherical acetabular shell and Modular Dual Mobility^®^ (MDM^®^) metal liner (Stryker Orthopaedics, Mahwah, NJ, USA) were used as the cup in all 33 hips. The MDM uses a shell with screw holes for additional fixation and a modular highly polished metal liner which articulates with polyethylene femoral head. In our series, additional screws were used with press-fit fixation considering osteoporotic bone. Migration and/or loosening of the implant was not seen in the postoperative radiographic evaluation.

### Statistical analysis

A professional medical statistical consultant performed the statistical analyses with GraphPad Prism 6 software (GraphPad Co. Ltd., USA) and JMP software package version 11.2 (SAS Institute, Cary, NC, USA). Groups were compared using the analysis of variance (ANOVA) followed by the non-parametric Mann-Whitney *U*-test and Fisher’s exact test. Effect measures in the present study were the odds ratios (ORs) for decrease of walking ability and one-year mortality, as estimated using logistic regression models. Adjusted ORs were estimated using a logistic regression model with adjustment for potential confounders such as age, neuromuscular diseases with weakness, duration of surgery, perioperative blood loss and preoperative walking ability. Data were expressed as the mean ± standard deviation. Values of *p* < 0.05 were considered statistically significant.

## Results

### The BHA group versus the THA group via the DAA

**Figure 1.**
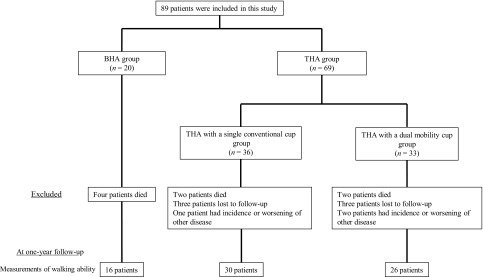
Study flowchart.

There were 20 patients in the BHA group and 69 patients in the THA group at the time of surgery (Figure 1, Table 1). The BHA group had a significantly shorter duration of surgery and significantly less perioperative blood loss compared with the THA group (Table 1). The intraoperative complications in the two BHA patients were calcar crack at the final repositioning, and two THA patients experienced a calcar crack during rasping of the femoral side (Table 1). In each case, the calcar crack was reinforced using wiring to the best extent possible. One of the two BHA patients had an infection and the other had periprosthetic fracture of the femur that required reoperation. One THA patient also had periprosthetic fracture of the femur; however, this was treated conservatively. The rate of reoperation was significantly higher in the BHA group than in the THA group (Table 1).

**Table 1.** Patient characteristics and peri- and post-operative outcomes.

Measurements	BHA	THA	*p* value	Single cup-THA	DMC-THA	*p* value
Patients (number)	20	69		36	33	
Age (years)	75.4 ± 7.9 (59–92)	77.5 ± 9.0 (56–95)	0.18^a^	75.2 ± 9.3 (56–91)	80.0 ± 7.9 (61–95)	0.019^a^
Sex (males/females)	4/16	15/54	1.0^b^	8/28	7/26	1.0^b^
Body mass index (kg/m^2^)	19.6 ± 3.3 (14.6–23.6)	21.2 ± 3.4 (13.4–29.1)	0.30^a^	21.2 ± 3.3 (15.9–28.6)	21.1 ± 3.5 (13.4–29.1)	0.77^a^
Operative side (right/left)	12/8	35/34	0.61^b^	22/14	13/20	0.093^b^
Neuromuscular disease with weakness, number (%) (cerebrovascular disorder/Parkinson’s disease)	2 (10%) (0/2)	10 (14.4%) (2/8)	1.0^b^	2 (5.5%) (2/0)	8 (24.2%) (0/8)	0.04^b^
Follow-up (months)	28.2 ± 26.6 (1.5–78.9)	16.9 ± 10.7 (0.33–36.7)	0.30^a^	17.9 ± 10.9 (1.8–36.7)	15.8 ± 10.6 (0.3–36)	0.35^a^
Duration of surgery (minutes)	89.4 ± 30.6 (63–160)	104.2 ± 21.4 (62–163)	0.0031^a^	100.4 ± 18.7 (62–151)	108.3 ± 23.6 (63–163)	0.19^a^
Perioperative blood loss (mL)	148.2 ± 201.0 (10–900)	398.8 ± 230.9 (20–930)	< 0.0001^a^	322.3 ± 248.7 (20–930)	480.0 ± 181.0 (31–920)	0.0012^a^
Intraoperative complications, number (%)	2 (10%)	2 (2.9%)	0.21^b^	0	2 (6.0%)	0.22^b^
Dislocation, number (%)	0	0	–	0	0	–
Postoperative complications, number (%)	2 (10%)	1 (1.45%)	0.12^b^	0	1 (3.0%)	0.47^b^
Reoperation, number (%)	2 (10%)	0	0.049^b^	0	0	–

Mean ± standard deviation (range);

a Mann-Whitney test;

b Fisher’s exact test;

*p* < 0.05: significant difference; BHA: bipolar hemiarthroplasty; THA: total hip arthroplasty; Single cup-THA: THA with a single conventional cup; DMC-THA: THA with a dual mobility cup.

The analysis of walking ability was performed after excluding patients who had died, were lost to follow-up or had occurence or worsening of other diseases during follow-up. In the THA group, 13/69 patients were excluded because of the inability to walk owing to cerebral haemorrhage, worsening malignant rheumatoid arthritis or cerebral infarction. Hence, the walking ability was assessed in 16 patients in the BHA group and 56 patients in the THA group (Figure 1). There was no significant difference between groups regarding pre- and post-operative walking ability (Table 2). After adjustment for covariates, multivariable logistic regression analysis also demonstrated no significant association with decrease of walking ability in either BHA or THA group (Table 3).

**Table 2.** Pre- and post-operative walking ability.

	BHA (*n* = 16)	THA (*n* = 56)	*p* value	Single cup-THA (*n* = 30)	DMC-THA (*n* = 26)	*p* value
Preoperative walking ability						
Category 1	14 (87.5%)	52 (92.1%)	0.33^a^	28 (93.3%)	24 (92.3%)	0.56^a^
Category 2	0	2 (3.5%)		1 (3.3%)	1 (3.8%)	
Category 3	2 (12.5%)	1 (1.7%)		0	1 (3.8%)	
Category 4	0	1 (1.7%)		1 (3.3%)	0	
Postoperative walking ability						
Category 1	12 (75%)	45 (80.3%)	0.54^a^	24 (80.0%)	21 (80.7%)	0.91^a^
Category 2	0	5 (8.9%)		3 (10.0%)	2 (7.6%)	
Category 3	4 (25%)	3 (5.3%)		2 (6.6%)	1 (3.8%)	
Category 4	0	3 (5.3%)		1 (3.3%)	2 (7.6%)	

Number (%);

a Mann-Whitney test;

*p* < 0.05: significant difference. Category 1: unaided walking, including walking using a T-cane; Category 2: walking using two crutches, including walkers for the elderly; Category 3: walking alongside a support (assisted walking); Category 4: use of a wheelchair; BHA: bipolar hemiarthroplasty; THA: total hip arthroplasty; Single cup-THA: THA with a single conventional cup; DMC-THA: THA with a dual mobility cup.

**Table 3.** Crude and adjusted odds ratio for decrease of walking category and one-year mortality, stratified by the type of arthroplasty via the direct anterior approach.

Decrease of walking category						
Arthroplasty	Number of subjects	Number of decrease of walking category (%)	OR [95% CI]	*p* value	Adjusted OR [95% CI]	*p* value
BHA	16	2 (12.5)	Ref	–	Ref	–
THA	56	8 (14.2)	1.22 [0.26–8.69]	0.80	1.12 [0.15–11.2]	0.90
Single cup-THA	30	4 (13.3)	Ref	–	Ref	–
DMC-THA	26	4 (15.3)	1.13 [0.24–5.27]	0.87	0.66 [0.072–4.7]	0.69

One-year mortality						

Arthroplasty	Number of subjects	Number of one-year mortality (%)	OR [95% CI]	*p* value	Adjusted OR [95% CI]	*p* value

BHA	20	4 (20)	Ref	–	Ref	–
THA	63	4 (6.2)	0.26 [0.057–1.23]	0.089	0.088 [0.0007–0.69]	0.020
Single cup-THA	34	2 (5.8)	Ref	–	Ref	–
DMC-THA	30	2 (6.6)	1.14 [0.13–10.0]	0.89	0.73 [0.045–10.1]	0.81

*p* < 0.05: significant difference; OR: odds ratio; CI: confidence interval; Adjusted: Arthroplasty was adjusted for age, neuromuscular diseases with weakness, duration of surgery, perioperative blood loss and preoperative walking ability; BHA: bipolar hemiarthroplasty; THA: total hip arthroplasty; Single cup-THA: THA with a single conventional cup; DMC-THA: THA with a dual mobility cup.

The analysis of one-year mortality was conducted on data from 20 patients in the BHA group, and 63 patients in the THA group, after excluding six patients who were lost to follow-up within the first postoperative year (Figure 1). After adjustment for covariates, multivariable logistic regression analysis demonstrated a significant association with one-year mortality in both BHA and THA groups [THA 0.088 (95% CI 0.0007–0.69); *p* = 0.020] (Table 3).

### The Single cup-THA group versus the DMC-THA group via the DAA

At the time of surgery, there were 36 patients in the Single cup-THA group and 33 in the DMC-THA group (Figure 1, Table 1). With respect to patient characteristics, the DMC-THA group had significantly greater age and more patients with neuromuscular diseases with weakness compared with the Single cup-THA group (Table 1). Two of 36 patients (5.5%) in the Single cup-THA group had cerebral infarction, but the symptoms of paralysis were barely noticeable. Eight of 33 patients (24.2%) in the DMC-THA group had Parkinson’s disease that was being treated with medication. There was no significant difference between the Single cup-THA and the DMC-THA groups regarding the duration of surgery (Table 1). The Single cup-THA group experienced significantly less perioperative blood loss than the DMC-THA group (Table 1).

The analysis of walking ability was performed after excluding patients who had died, were lost to follow-up or had incidence or worsening of other diseases during follow-up. In the Single cup-THA group, one patient was excluded because of cerebral haemorrhage that caused the inability to walk. In the DMC-THA group, two patients were excluded because they could not walk owing to other medical diseases such as worsening rheumatoid arthritis and cerebral infarction. Hence, the walking ability was assessed in 30 patients in the Single cup-THA group and 26 patients in the DMC-THA group (Figure 1). There was no significant difference between groups regarding pre- and post-operative walking ability (Table 2). After adjustment for covariates, multivariable logistic regression analysis also demonstrated no significant difference in the decrease of walking ability in either the Single cup-THA or the DMC-THA group (Table 3).

The analysis of one-year mortality was performed after excluding patients lost to follow-up within one year postoperatively. Hence, one-year mortality was assessed in 33 patients in the Single cup-THA group and 30 patients in the DMC-THA group (Figure 1). After adjustment for covariates, multivariable logistic regression analysis also demonstrated no significant association with one-year mortality in either the Single cup-THA or the DMC-THA group (Table 3).

## Discussion

Although THA for displaced femoral neck fracture reportedly results in superior pain relief, function and a lower reoperation rate than BHA, the dislocation rate after THA is higher than that after BHA [2]. The present study demonstrates that THA via the DAA for displaced femoral neck fracture resulted in almost no postoperative decrease in walking ability and few serious adverse events, with less risk of dislocation, which was previously a weakness of THA compared with BHA. Furthermore, the present study also demonstrates the safety and effectiveness of THA via the DAA with a DMC for displaced femoral neck fracture for the older patients and those with neuromuscular diseases with weakness.

Although patients who underwent THA via the DAA experienced increased intraoperative blood loss associated with longer duration of surgery compared with those who underwent BHA via the DAA, this did not increase the number of complications or the mortality rate. Blomfeldt et al. reported a longer duration of surgery and increased intraoperative blood loss in THA compared with BHA, but they also found no significant differences between the groups regarding complications or mortality [15]. The present study found no postoperative decrease in walking ability in more than 85% of patients at one year after the operation in all groups. A systematic review reported that THA was superior to BHA regarding functional outcomes such as the Harris Hip Score and pain relief [2]. Blomfeldt et al. also reported that THA provides better function than BHA as soon as one year postoperatively, without increasing the complication rate [15]. The present study found that the THA group had lower one-year mortality than the BHA group using a multivariable logistic regression analysis. Although several meta-analyses have found no difference in one-year mortality between those undergoing BHA versus THA [2, 3, 16], Avery et al. reported that at 100 months postoperatively a significantly greater proportion of BHA patients than THA patients had died [19].

THA via the DAA with a DMC for displaced femoral neck fracture was effective in reducing the dislocation and maintaining mobility, with less concern about complications in patients with a high risk of dislocation such as greater age or neuromuscular diseases with weakness. A systematic review pooling the data of 780 patients and 47 events (6%) revealed a significant risk of dislocation after treatment for dislocated femoral neck fractures with THA compared with BHA [2]. Thürig et al. reported that dislocation occurred in 2/86 patients (2.3%) with THA via the DAA, which was a lower rate than in other studies [18]. Several studies reported higher dislocation rates for patients with neuromuscular or cognitive disorders and greater age [20, 21]. Park et al. also reported that THA using large-diameter metal-on-metal articulation in patients with neuromuscular weakness, can produce satisfactory outcomes with early functional recovery and a low dislocation rate [17]. However, THA using large-diameter metal-on-metal articulation is associated with some problems such as metal ion concentration and pseudotumors [22, 23]. Constrained cups also better prevent dislocation than standard cups but with an increased loosening rate [24]. The reason for more perioperative blood loss in the DMC-THA group than in the Single cup-THA group might be that the additional soft tissue release was demanded in the case that intraoperative dislocation with larger head of trial DMC implant was difficult due to high soft tissue tension by using DAA [9].

There were several limitations to this study. First, the strength of our results is limited, as the study was a retrospective with a small number of subjects. Although the power calculation that was conducted before the study started was based on a meta-analysis of randomized controlled trials (RCTs), it was logistically difficult to collect such calculated numbers in this retrospective two centre study. Second, the follow-up periods were limited. We are aware that longer follow-up is needed in order to identify clinical outcomes about each treatment. However, this fracture has effects on the quality of life for older patients in particular. So it is also important for them to maintain their pre-injury activity level after the operation and to prevent the occurrence of complications such as disuse syndrome in the short term. Third, comorbid diseases other than neuromuscular disease with weakness could not be estimated. Although the patients were randomly allocated to two centres, a selection bias might exist among each group. In the future, these issues will require further investigation.

## Conclusion

THA via the DAA is one of the best treatments for displaced femoral neck fracture, with a low risk of dislocation. Furthermore, THA via the DAA with a DMC is a safe and effective for treating displaced femoral neck fracture in patients with a high risk of dislocation.

## Conflict of interest

The authors declare that they have no conflict of interest.
